# *GsERF1* enhances *Arabidopsis thaliana* aluminum tolerance through an ethylene-mediated pathway

**DOI:** 10.1186/s12870-022-03625-6

**Published:** 2022-05-24

**Authors:** Lu Li, Xingang Li, Ce Yang, Yanbo Cheng, Zhandong Cai, Hai Nian, Qibin Ma

**Affiliations:** 1 State Key Laboratory for Conservation and Utilization of Subtropical Agro-bioresources, South China Agricultural University, Guangzhou, Guangdong 510642 People’s Republic of China; 2 Key Laboratory of Plant Molecular Breeding of Guangdong Province, College of Agriculture, South China Agricultural University, Guangzhou, Guangdong 510642 People’s Republic of China; 3 Guangdong Subcenter of the National Center for Soybean Improvement, College of Agriculture, South China Agricultural University, Guangzhou, Guangdong 510642 People’s Republic of China; 4 Guangdong Provincial Laboratory of Lingnan Modern Agricultural Science and Technology, South China Agricultural University, Guangzhou, Guangdong 510642 People’s Republic of China; 5 Zengcheng Teaching and Research Base, South China Agricultural University, Guangzhou, Guangdong 510642 People’s Republic of China

**Keywords:** AP2/ERF family, *GsERF1*, Transcription factor, ET, Aluminum stress, ACS

## Abstract

Ethylene response factor (ERF) transcription factors constitute a subfamily of the AP2/ERF superfamily in plants and play multiple roles in plant growth and development as well as in stress responses. In this study, the *GsERF1* gene from the wild soybean BW69 line (an Al-resistant *Glycine soja* line) was rapidly induced in response to aluminum stress. Quantitative real-time PCR (qRT–PCR) analysis showed that the *GsERF1* gene maintained a constitutive expression pattern and was induced in soybean in response to aluminum stress, with increased amounts of transcripts detected in the roots. The putative GsERF1 protein, which contains an AP2 domain, was located in the nucleus and maintained transactivation activity. In addition, under AlCl3 treatment, GsERF1 overexpression increased the relative growth rate of the roots of Arabidopsis and weakened the hematoxylin staining of hairy roots. Ethylene synthesis-related genes such as *ACS4*, *ACS5* and *ACS6* were upregulated in *GsERF1* transgenic lines compared with the wild type under AlCl_3_ treatment. Furthermore, the expression levels of stress/ABA-responsive marker genes, including *ABI1, ABI2, ABI4, ABI5* and *RD29B*, in the *GsERF1* transgenic lines were affected by AlCl_3_ treatment, unlike those in the wild type. Taken together, the results indicated that overexpression of *GsERF1* may enhance aluminum tolerance of *Arabidopsis* through an ethylene-mediated pathway and/or ABA signaling pathway, the findings of which lay a foundation for breeding soybean plants tolerant to aluminum stress.

**Supplementary Information:**

The online version contains supplementary material available at 10.1186/s12870-022-03625-6.

## Introduction

Heavy metal toxicity, such as aluminum (Al) toxicity, is a major limiting factor for crop production worldwide [1]. When the pH of the soil is lower than 5.0, aluminum is present in an ionic form, i.e., Al^3+^, which strongly inhibits root growth and function, reducing crop yields [2]. Plant species and varieties vary widely in their ability to tolerate aluminum toxicity. Some plant species or varieties have evolved high levels of tolerance mechanisms to survive in acidic soils. Wild soybean has been growing in acidic soils in South China for a long time, and as such, there resources available that can provide tolerance, which plays an important role in improving the stress resistance of soybean [3]. Transcription factors are involved in stress responses; transcription factors from the WRKY, bZIP and NAC families have been shown to participate in the aluminum stress response and to regulate the aluminum tolerance of plants [4–6]. However, the involvement of the ERF transcription factor family in the aluminum stress response has not been reported. Plants employ a complex regulatory network to cope with a variety of stresses during growth and development. A variety of plant hormones play important roles from the beginning of sensing stress signals to the response of plants to stress. Under normal circumstances, the ethylene content in plants is maintained at a low level. However, plant ethylene content changes in response to biological stress or abiotic stress. The response of ethylene production after stress stimulation is transmitted through corresponding signal transduction pathways, which can regulate downstream genes, causing a series of reactions in plant cells and an associated response to stress [7–9]. Previous studies have shown that when plants are subjected heavy metal toxicity, the general response involves increased production of ethylene. For example, plants increase their production of ethylene under toxic levels of cadmium (Cd), copper (Cu), iron (Fe), nickel (Ni) and zinc (Zn). Moreover, it has been found that the change in ethylene under heavy metal stress is due to the increased expression of ethylene-related biosynthesis-related genes and/or changes in the expression of ethylene-responsive genes. Regarding these changes, it has been found that the increase in ethylene during stress can have negative effects on plants. However, ethylene can alleviate the inhibition of the photosynthetic capacity of mustard under cadmium stress. These findings suggest that ethylene involves a complex two-way regulatory function under stress, which depends on its endogenous level [10–13].

ERF transcription factors (ethylene response factors) constitute a subfamily of the AP2/ERF superfamily and can be divided into three categories according to the number of AP2/ERF domains: AP2, ERF and RAV [14]. The ERF family protein members contain an AP2/ERF domain consisting of 58–60 highly conserved amino acids, which constitutes the main functional region of ERF family proteins [15]. Ethylene response factors (ERFs) not only play important roles in plant growth and development but also play very important roles in the plant response to stress [15]. Previous studies have shown that ERF family genes are involved in plant growth and development in rice, Arabidopsis and other plant species. For example, *OsERF1* is constitutively expressed in different organs of rice and is upregulated by ethylene. Overexpression of *OsERF1* significantly affects the growth and development of transgenic Arabidopsis by promoting the expression of the ethylene-responsive genes *PDF1.2* and β-chitinase [16]. AtERF71/HRE2 can activate the expression of downstream genes by binding the motifs of GCC boxes and DRE/CRT elements, regulate the expansion of root cells and play important roles in root development [17]. Julien Pirrello found that overexpression of the *Sl-ERF2* gene in transgenic tomato lines can lead to early seed germination and enhanced hypocotyl formation in dark-grown seedlings. Recently, the transcription factor ERF139 was found in poplar to regulate the expansion of xylem cells and the deposition of secondary cell walls [18].

In recent years, an increasing number of ERF family genes have been found to function in stress tolerance in plants. Under drought stress, overexpression of the rice genes *OsERF71*, *OsERF101* and *OsERF48* was shown enhance the drought resistance of rice [19–22]. Heterologous overexpression of the soybean gene *GmERF3* can enhance tobacco drought resistance [23], and overexpression of *AtERF019* can enhance drought resistance in Arabidopsis [24]. Overexpression of *GmERF135* can enhance the salt tolerance of Arabidopsis plants under salt stress. Moreover, *GmERF135* can promote the growth of transgenic hairy roots under salt stress [25]. In wheat, overexpression of *ERF1*-V can enhance the salt tolerance of wheat, and heterologous overexpression of *GmERF7* can enhance the salt tolerance of tobacco [26, 27]. Under alkaline stress conditions, heterologous overexpression of *GsERF71* and *GsERF6* from wild soybean and *VaERF3* from red bean plants can enhance the resistance of Arabidopsis to alkali stress [28–30], and overexpression of *ZmEREB180* in maize can enhance maize submergence tolerance [31]. Heterologous overexpression of *VaERF092* and *ERF105* also enhance Arabidopsis cold tolerance [32, 33]. Moreover, overexpression of *GmERF75* in Arabidopsis can enhance the osmotic stress tolerance of Arabidopsis, and GmERF75 can promote osmotic stress tolerance in transgenic hairy roots [34]. ERF genes can also enhance plant resistance to pathogens. *AtERF14* was found to regulate the plant defense response [35]. In Arabidopsis, *ERF11* and *ERF15* can positively regulate immunity to *Pseudomonas syringae* [36, 37], and in soybean, *GmERF13* and *GmERF5* can enhance resistance to *Phytophthora sojae* [38, 39].

However, few ERF family genes have been reported to be involved in the response of plants to aluminum stress. In previous studies, the *GsERF1* gene was found to be rapidly induced in response to aluminum stress in the wild soybean line BW69 (an Al-resistant *Glycine soja* line) [3]. However, it is unclear whether *GsERF1* is involved in regulating aluminum tolerance in plants. Therefore, the function of *GsERF1* was further investigated to elucidate its involvement in the mechanism underlying tolerance to aluminum stress.

## Results

### Isolation and sequence analysis of the *GsERF1* gene

**Fig. 1:**
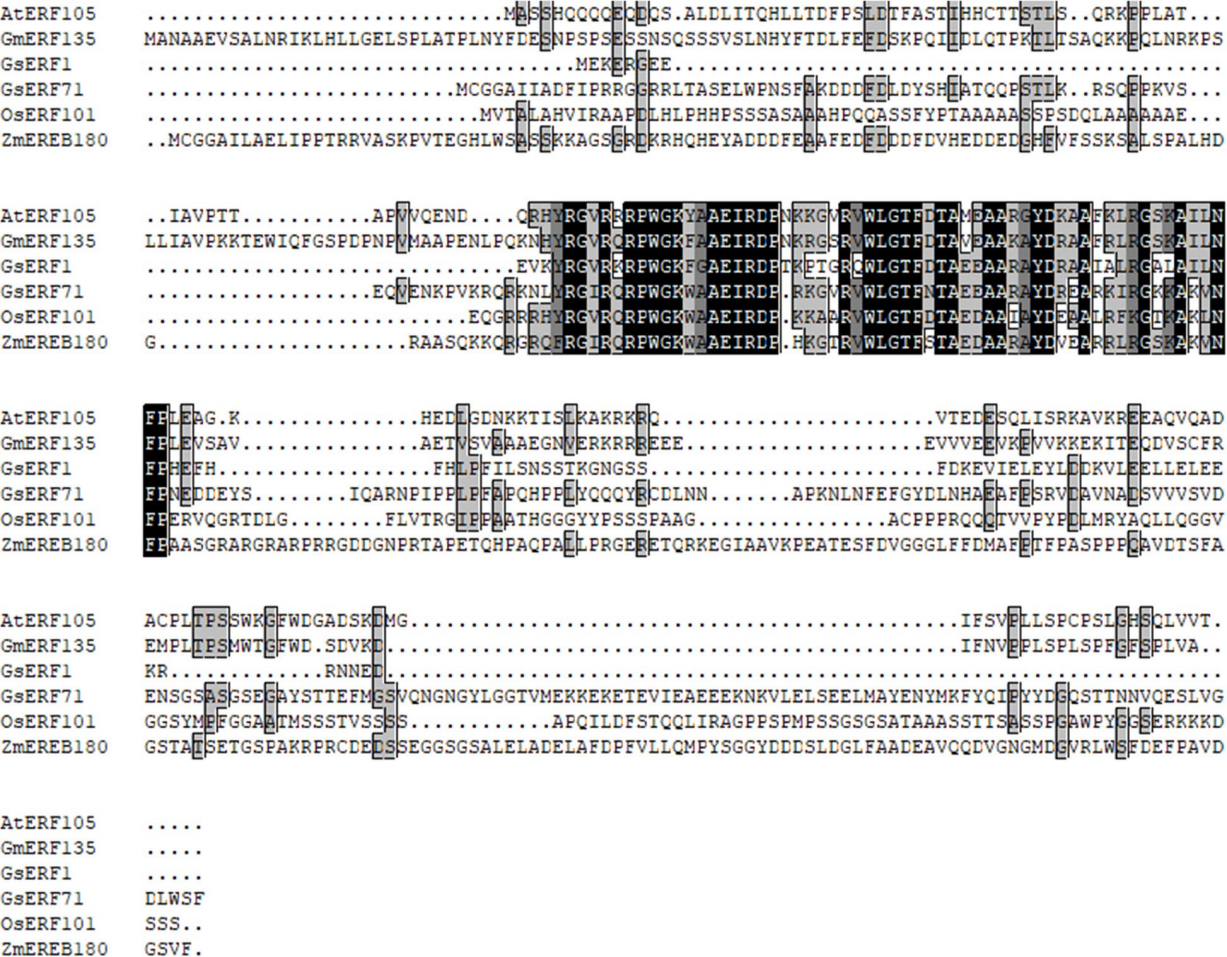
Sequence alignment of the AP2 domain. The shaded part of the figure indicates the AP2 domain. The protein sequences of the selected ERF genes were obtained from Phytozome or GenBank, and the included genes and their accession numbers are as follows: *AtERF105* (NP_568755.1), *GmERF135* (Glyma.17G145300), *OsERF71* (XP_015643752.1), *OsERF101* (Os04g32620), and *ZmEREB180* (NC_024459.2). The sequence alignment was carried out by DNAMAN software

**Fig. 2:**
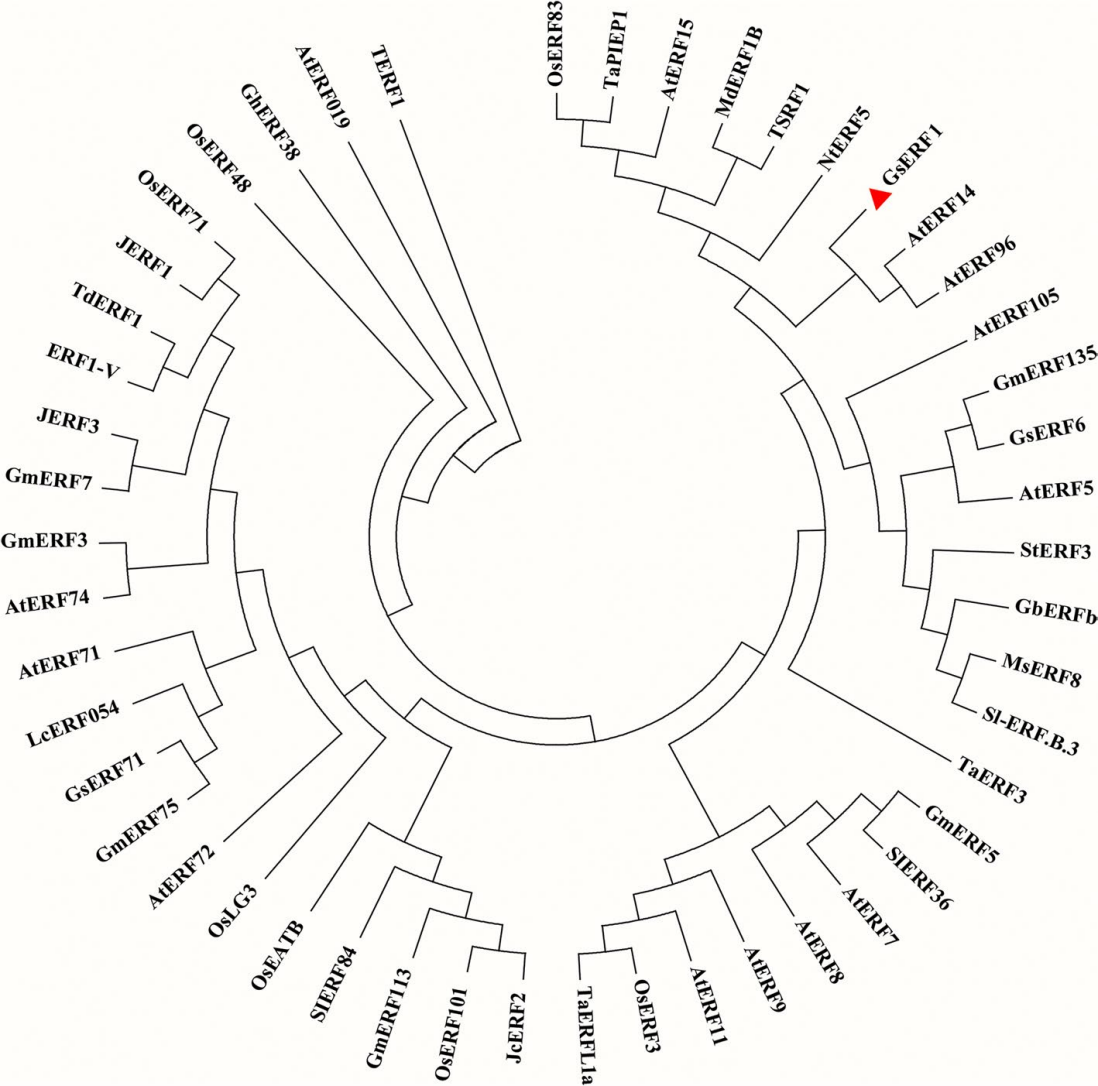
Phylogenetic relationships among 48 transcription factors of the ERF family associated with stress resistance. The protein sequences of the selected ERF genes were obtained from Phytozome or GenBank, and the accession numbers are shown in the supplementary material

In this study, the full-length cDNA sequence of the *GsERF1* gene was cloned from the BW69 line *Glycine soja,* which is tolerant to Al toxicity. The primers used were designed according to the homologous *GsERF1* gene in *Glycine max*, *Glyma09g52900*. The *GsERF1* gene consists of a complete open reading frame (ORF) of 369 bp, the sequence of which is 99% identical to that of *Glyma09g52900* based on the Phytozome genome database, and encodes a protein of 122 amino acids. The predicted GsERF1 protein contains a conserved DNA-binding domain (AP2/ERF domain) of 58 amino acids, which is reported to be the primary functional region. Alignment analysis revealed that the GsERF1 protein was 68 to 97% similar to other proteins encoded by homologous genes and had similar domains (Fig. 1). The analysis of the ERF gene family indicated that GsERF1 is a member of the B-2 subgroup [14]. Many ERF family genes have been reported to have similar functions and play roles in the response to both biotic and abiotic stresses in plants. Phylogenetic analysis showed that GsERF1 and GmERF5 are closely related and belong to the same branch (Fig. 2).

### Analysis of *GsERF1* expression patterns

**Fig. 3:**
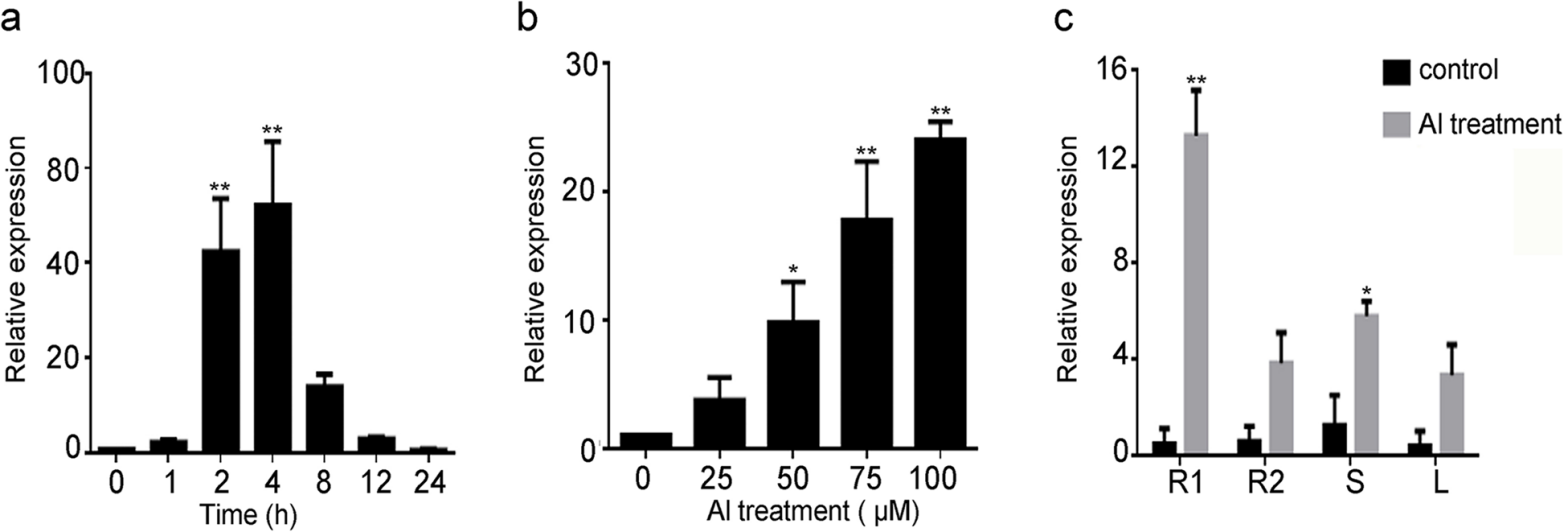
Expression patterns of *GsERF1* in different tissues of plants under AlCl_3_ treatment. **a ***GsERF1* expression in the roots exposed to 30 μM AlCl_3_ (0.5 mM CaCl_2_, pH 4.5) for 0 to 24 h. **b ***GsERF1* expression in the roots exposed to 0 to 100 μM AlCl_3_ (0.5 mM CaCl_2_, pH 4.5) for 6 h. **c ***GsERF1* expression in soybean root apices (R1, 0–1 cm; R2, 0–2 cm), stems (S) and leaves (L) in the absence or presence of Al stress. The values are the means ± SDs (*n* = 3). The asterisks show significant differences between the control and Al treatments according to Student’s t test: *, *P* < 0.05; **, *P* < 0.01

Quantitative real-time PCR (qRT–PCR) was performed to assess the transcript levels of *GsERF1* in BW69 plants. The qRT–PCR results showed that *GsERF1* was constitutively expressed in the roots, stems and leaves. Under aluminum stress, the expression level of *GsERF1* in the roots, stems and leaves was significantly increased; this was the case especially in 1-cm-long root tips, in which the expression level increased by 13-fold (Fig. 3c). Over time, *GsERF1* was rapidly induced in response to aluminum stress, and the transcripts of *GsERF1* reached their maximum level at 4 h, after which the mRNA transcripts of *GsERF1* began to decline (Fig. 3a). Under treatment with different concentrations of aluminum, *GsERF1* transcription increased with increasing AlCl_3_ concentration. When the concentration of AlCl_3_ was 100 μM, the level of *GsERF1* mRNA was 25 times that of the control (Fig. 3b).

### GsERF1 is a nuclear protein with transactivation activity

**Fig. 4:**
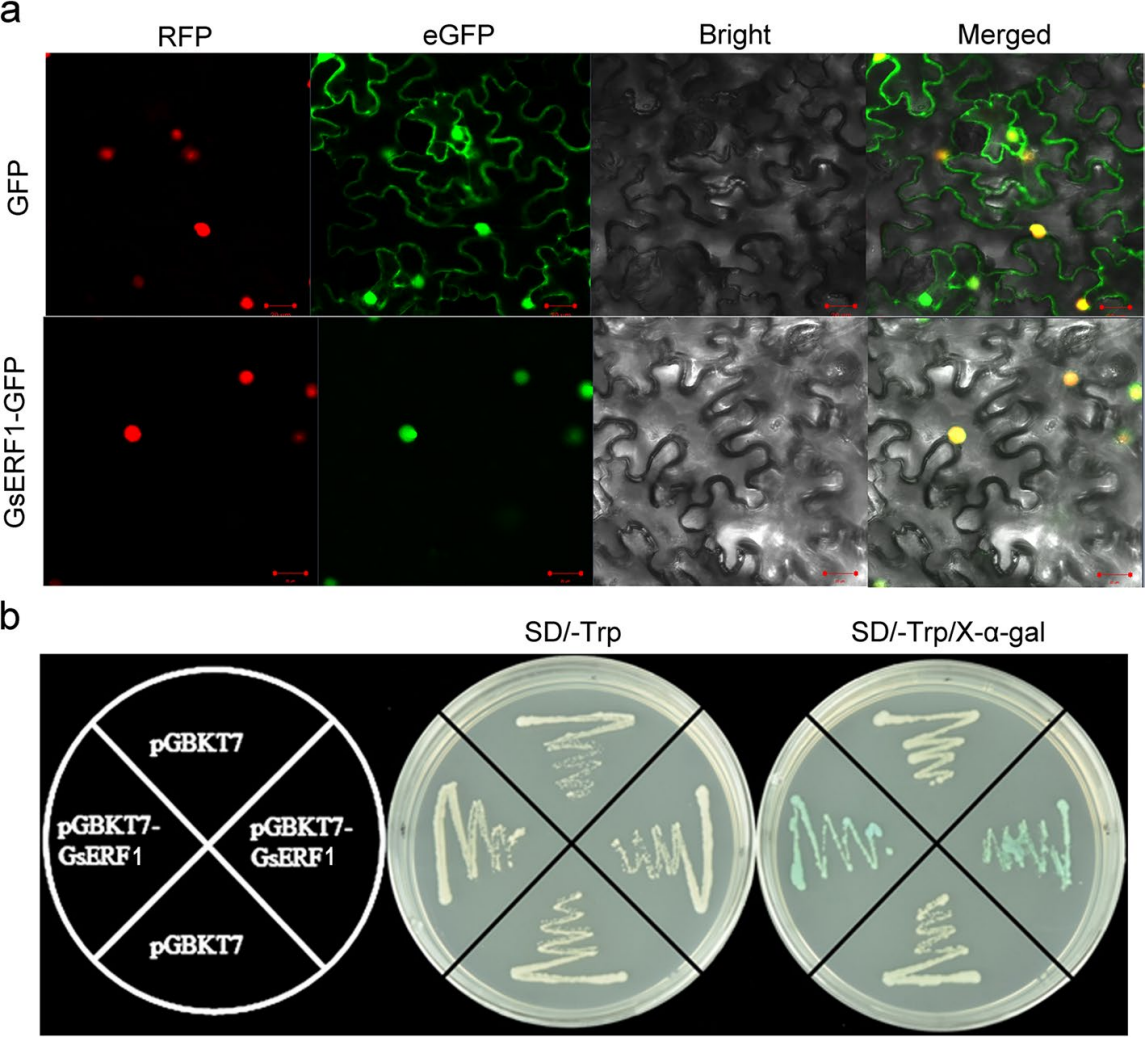
GsERF1 proteins localize to the nucleus and exhibit transactivation activity. **a** Nuclear localization of the GsERF1 protein in leaf epidermal cells of *Nicotiana benthamiana*. Nicotiana leaves transiently expressing GFP alone (upper) and GsERF1-GFP (bottom) proteins were observed with a confocal microscope (Olympus FluoView FV1000, Japan). **b** Transactivation assay of truncated GsERF1 proteins. The full-length GsERF1 sequence was fused to the GAL4 DNA-binding domain and then expressed in yeast strain Y2H gold. The transformed yeast cells were plated and grown on control medium plates (SD/−Trp) or selective medium plates (SD/−Trp + X-α-gal)

To determine the cellular localization of the GsERF1 protein, its localization was analyzed by expressing a gene encoding a GsERF1-eGFP fusion protein under the control of the CaMV35S promoter in tobacco epidermal cells. An empty vector (pCAMBIA1302-eGFP) was used as a control. As shown in Fig. 4a, the GFP fluorescence was distributed throughout the whole cells, while the GsERF11-eGFP fusion protein fluorescence was visible only in the cell nuclei. These results clearly indicated that GmERF5 is a nuclear localized.

Yeast two-hybrid analysis was used to determine whether GsERF1 could act as a transcriptional activator. The full-length *GsERF1* gene was fused to the GAL4 DNA-binding domain and then expressed in the yeast strain Y2H gold to measure transcriptional activation activity; the yeast cells were grown on control medium plates (SD/−Trp) or selective medium plates (SD/−Trp + X-α-gal). Yeast cells containing pGBKT7 plasmids with only the GAL4 DNA-binding domain were used as negative controls. The results showed that only yeast colonies carrying GsERF1 could activate the expression of the reporter gene and cause the colonies to appear blue on the selective medium plate (Fig. 4b).

### Overexpression of *GsERF1* enhanced plant Al tolerance

**Fig. 5:**
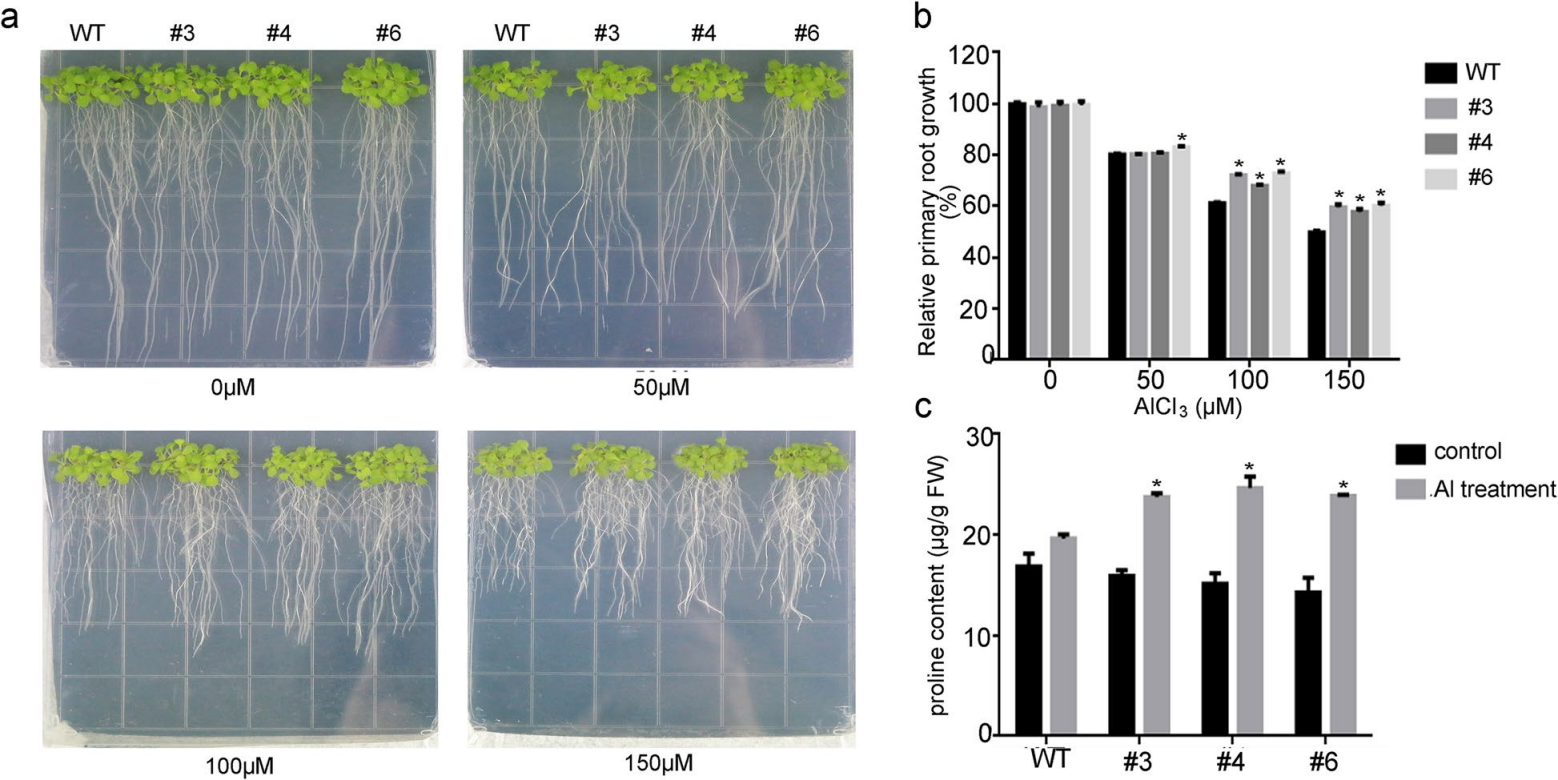
*GsERF1* enhanced the resistance of Arabidopsis plants to Al stress. **a** Root growth of wild-type and *GsERF1*-overexpressing Arabidopsis with or without Al treatment. **b** Relative root growth was calculated. **c** Free proline contents in wild-type and *GsERF1*-overexpressing Arabidopsis. WT: wild type. #3, #4 and #6: T_3_ generation *GsERF1* lines. The data are mean values ± SDs (*P < 0.05; Student’s t test). All the experiments included three biological replications

To investigate the effect of *GsERF1* under aluminum stress, GsERF1 was overexpressed in Arabidopsis to obtain transgenic lines (Fig. S1). Then, three homozygous lines with high expression were selected for phenotypic identification. Under AlCl_3_ treatment_,_ the growth of the *GsERF1* transgenic plants and WT plants was significantly inhibited, but the root growth of the *GsERF1* overexpression (OX) lines was less inhibited than that of the WT plants was. The statistical results also showed that the relative root growth of the *GsERF1* transgenic plants was significantly higher than that of WT plants (Fig. 5a&b). Similarly, the fresh weight of *GsERF1* transgenic plants was greater than that of the WT plants (Fig. S2). The proline content of the transgenic plants and wild-type plants increased after aluminum treatment, but the proline content in the *GsERF1* transgenic plants was much higher than that in the wild-type plants (Fig. 5c).

**Fig. 6:**
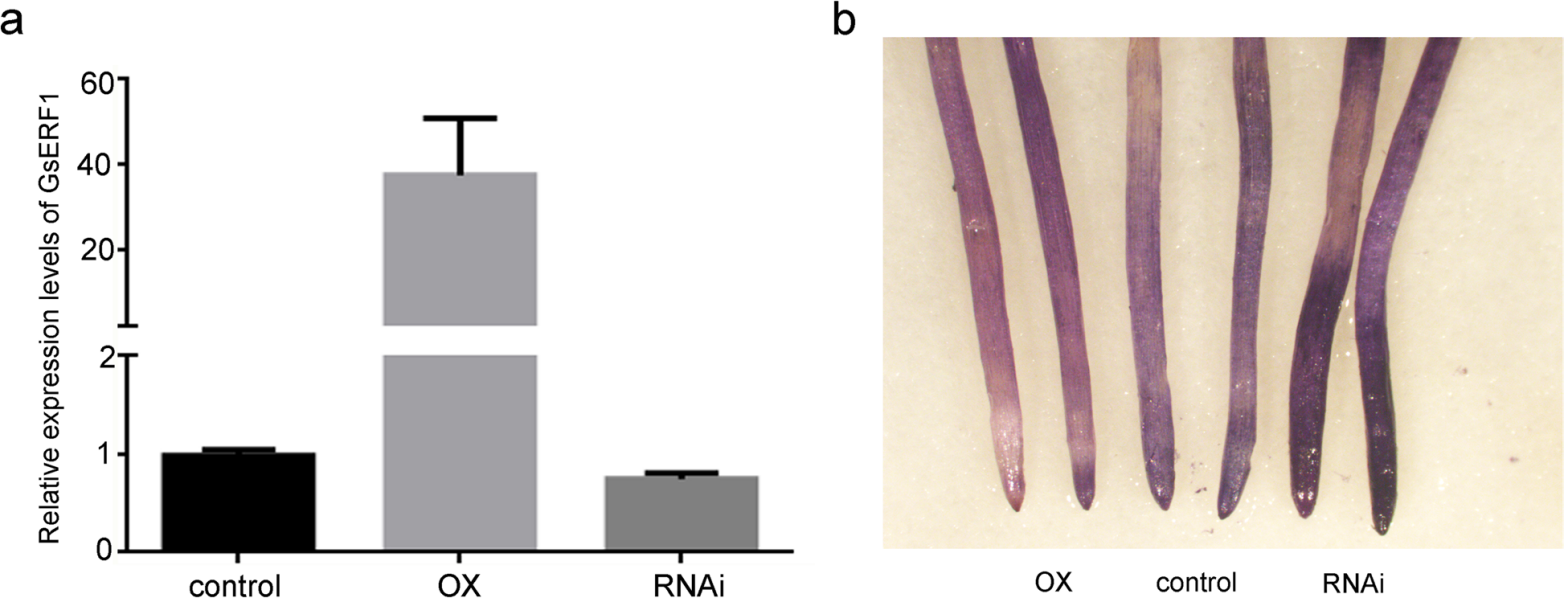
Root tip staining phenotypes of hairy roots. **a** Expression levels of *GsERF1* in control, OX-GsERF1 and RNAi-GsERF1 hairy roots. **b** The control, OX-GsERF1 and RNAi-GsERF1 hairy roots were stained with hematoxylin after Al treatment. The control, OX-GsERF1 and RNAi-GsERF1 hairy roots were subjected to 50 μM AlCl_3_ (0.5 mM CaCl_2_, pH 4.5) for 12 h. OX: *GsERF1-*overexpressing plants; RNAi: *GsERF1-*silenced plants. The data are the mean values ± SDs, and all the experiments included three biological replications

To verify the role of *GsERF1* in soybean, we generated hairy root lines in which the gene was silenced through soybean hairy root transformation involving RNAi-interference. We detected that GsERF1 mRNA levels were 38 times higher in the overexpression lines than in the WT. However, *GsERF1* mRNA levels were 80% lower in the RNAi lines than in the WT lines (Fig. 6a). To visualize activity of GsERF1, the hairy roots of soybean were stained with hematoxylin after treatment in a solution consisting of 50 μM AlCl_3_ for 6 hours. The staining showed that the hairy roots of the control displayed stronger staining than did those of the OX-GsERF1 transgenic lines but weaker staining than those of the RNAi-GsERF1 transgenic lines (Fig. 6b). These results indicated that the amount of Al^3+^ binding in the hairy roots was the lowest in the OX-GsERF1 lines, while the amount of Al^3+^ binding in the hairy roots was the highest in the RNAi-GsERF1 lines. Taken together, the staining results suggested that *GsERF1* overexpression can enhance the tolerance of Arabidopsis and soybean to aluminum stress.

### Determination of ethylene precursors

**Fig. 7:**
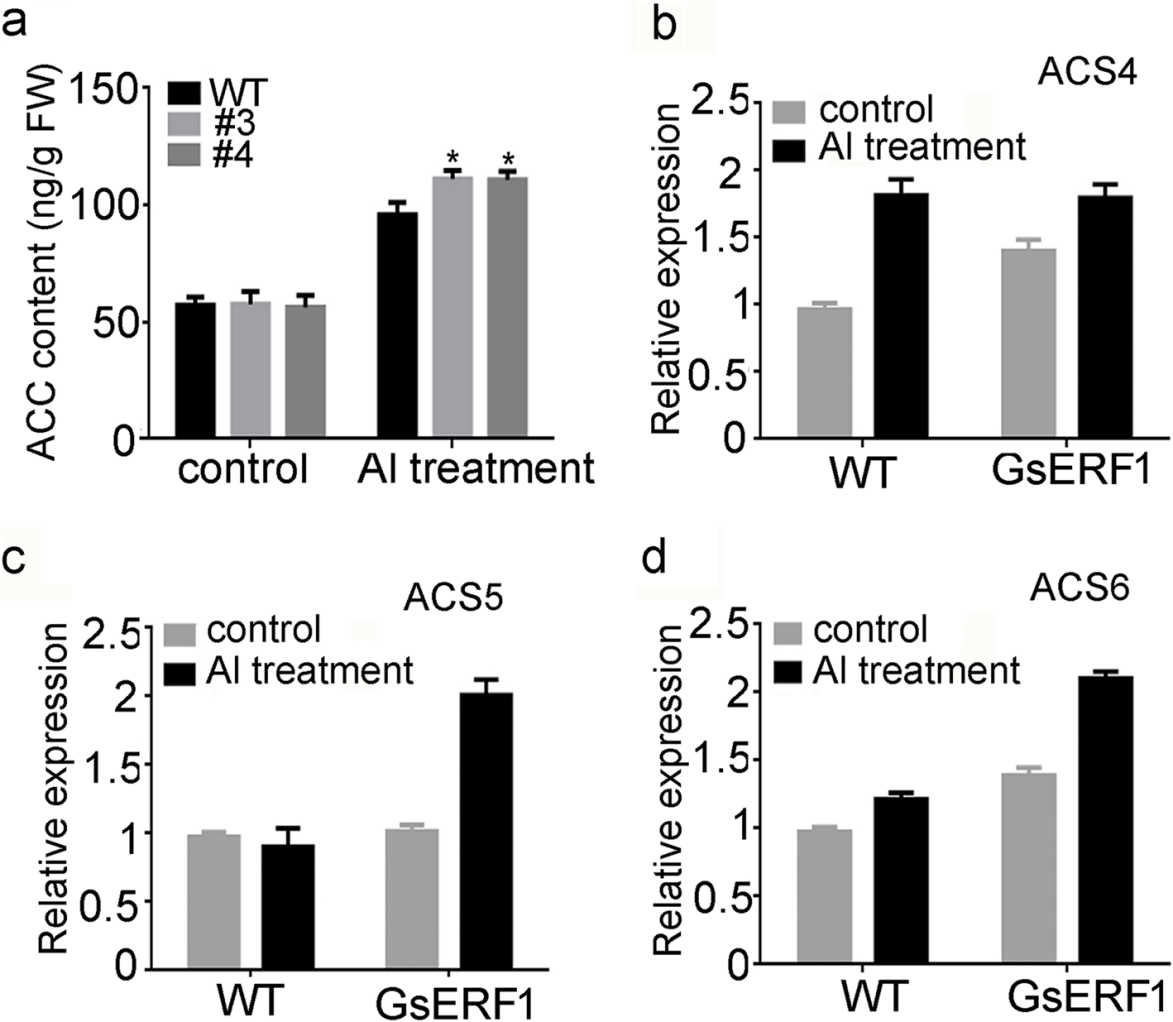
ACC content and the expression of ACC biosynthesis-related genes in *GsERF1*-overexpressing and wild-type Arabidopsis plants. **a** ACC contents in *GsERF1*-overexpressing and wild-type Arabidopsis plants under control and Al treatment conditions. **b-d** Real-time PCR analysis of the expression levels of ACC biosynthesis-related genes under control and Al treatment conditions. The samples were taken from three independent lines and equally mixed for quantitative analysis. The data are mean values ± SDs (*P < 0.05; Student’s t test). The error bars represent the standard errors of three replicates. WT: wild type. #3 and #4: T_3_-generation GsERF1 transgenic lines

To understand the molecular mechanism through which *GsERF1* is involved in tolerance to Al stress, several genes from ERF family members were evaluated to investigate their responses to Al stress, and the ACC (an ethylene precursor) content was determined in Arabidopsis. On the basis of *GsERF1* expression upregulated in response to ethylene (ET) (Fig. S3), the changes in ACC content in the *GsERF1* overexpression (OX) lines and wild-type (WT) Arabidopsis were determined after 10 days of aluminum treatment. The results showed that the ACC content in the *GsERF1*-overexpressing plants was higher than that in the wild-type plants, while there was little difference in ACC content between the wild-type plants and overexpression plants in the absence of aluminum treatment (Fig. 7a). These results suggested that ET signal transduction may be involved in the aluminum tolerance pathway induced by the *GsERF1* gene. In addition, the qRT–PCR results showed that the expression levels of the *ACS4*, *ACS5* and *ACS6* genes, which are involved ethylene synthesis, were significantly increased in the *GsERF1*-overexpressing plants compared with the wild-type plants under AlCl_3_ treatment (Fig. 7b, c&d). To verify whether ethylene is involved in the aluminum tolerance pathway, *GsERF1* overexpression lines and wild-type Arabidopsis were treated with ACC and AlCl_3_ together. However, the data showed that with the addition of ACC, the root elongation advantage of the transgenic lines no longer occurred in the presence of 100 μM AlCl_3_ (Fig. 9)_._ These results may imply that ethylene can enhance the tolerance of plants to aluminum stress. However, this hypothesis needs to be further verified by subsequent experiments.

## Discussion

Aluminum toxicity has a great influence on the roots of plants and directly affects crop yields. Therefore, it is important in theory and in practice to identify new aluminum tolerance genes and determine their functions. The AP2/ERF family is one of the largest families of plant transcription factors, the members of which are involved in many aspects of plant development and responses to multiple environmental stresses [40–43]. In this study, the GsERF1 gene, which encodes an ERF transcription factor, was isolated on the basis of the aluminum stress resistance gene expression profile of wild soybean line BW69 [3]. Sequence analysis showed that the GsERF1 protein has a highly conserved AP2 domain with characteristics typical of those of domains of the members of the B-3 subgroup of the ERF superfamily (Fig. 1) [14]. Furthermore, like many other ERF transcription factors, the GsERF1 protein localizes to the nucleus and has self-activation activity (Fig. 4). Therefore, we speculate that GsERF1 may play a role in plant biological processes.

According to previous studies, members of the AP2/ERF family have important functions in plants in response to various environmental conditions and at different growth and development stages. This is the case for various members of the B-3 subgroup: *OsERF48* can enhance the tolerance of plants to drought and salt stresses [19], *AtERF15* can promote the positive regulation of the immune response in Arabidopsis [37], *AtERF096* can reduce the water loss rate in Arabidopsis [44], NtERF5 enhances resistance to tobacco mosaic virus, and *GsERF16* regulates plant tolerance to bicarbonate in Arabidopsis [28]. ERF genes also play a key role in plant growth and development. For example, *MdERF1B* can regulate the biosynthesis of anthocyanins and procyanidins in applie [45], and *OsERF1* significantly affects the growth and development of transgenic Arabidopsis [16]. However, no ERF family genes have been reported to be involved in the response to Al stress in plants. In the present study, *GsERF1* was rapidly induced in response to aluminum stress, with the greatest amount of transcription occurring under AlCl_3_ treatment (Fig. 3). This result suggested that *GsERF1* may play a certain role in the response to aluminum stress.

In recent years, there have been an increasing number of reports in which soybean hairy root transformation was used to verify the function of soybean genes in tolerance to aluminum stress. Among these studies, hematoxylin staining of the GmME1-OE hairy root tips was found to be lighter with decreasing aluminum content. Moreover, overexpression of *GmGRPL* resulted in obvious aluminum resistance in transgenic lines compared with controls. Under aluminum stress, the aluminum contents in the hairy roots of *GmGRPL* overexpression lines were lower than those in the controls, and the former presented higher antioxidant capacity. All the above studies are based on the colorimetric properties of hematoxylin, and the degree of staining of hematoxylin in plants roots increased with increasing degree of toxicity of aluminum ions (acidic conditions). Therefore, visual evaluation of stained regions can be used to detect the accumulation of Al in plant roots [46]. In our study, hematoxylin staining was also used to verify whether the *GsERF1* gene functions in the tolerance to aluminum under acidic conditions. The results showed that the hairy roots of the *GsERF1-*overexpressing lines were less stained than were those of the wild type after aluminum treatment, while the *GsERF1-*RNAi lines were more stained than the wild type (Fig. 6). This indicates that the hairy roots of the GsERF1-overexpressing transformants have a lower aluminum content than those of the wild type do, while the GsERF1-RNAi lines have the highest root aluminum content. Previous studies have shown that root tip elongation is inhibited when plants are subjected to aluminum stress, so root tip elongation is one of the indicators of aluminum tolerance [47]. A similar phenomenon was also revealed by our results: the relative root growth of the *GsERF1* transgenic lines with a higher degree of proline accumulation was greater than that of the wild type (Fig. 5). Previous studies have indicated that the proline content increases after plants experience stress, while a large amount of proline can help plants reduce the damage caused by stress. Therefore, on the basis of our experimental results, we speculated that the *GsERF1* gene can enhance the tolerance of transgenic Arabidopsis to aluminum stress.

**Fig. 8:**
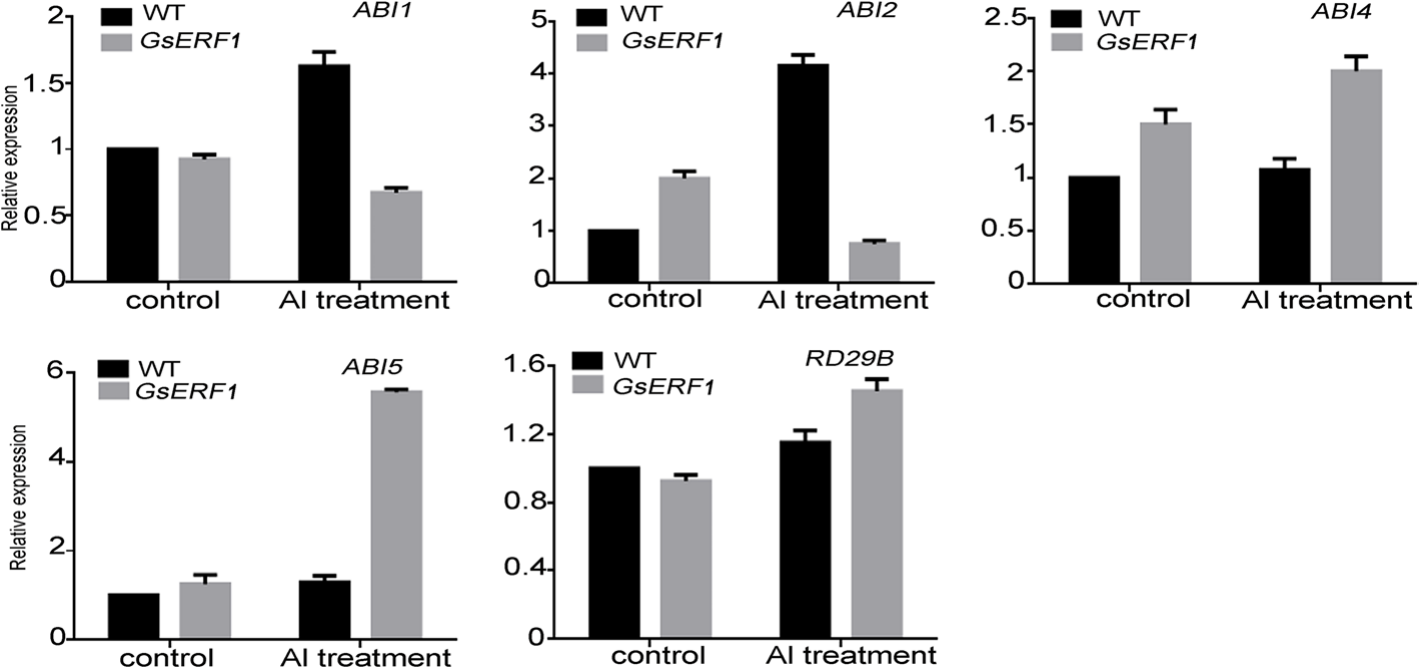
Expression levels of ABA transport-related genes in GsERF1-overexpressing and wild-type Arabidopsis. Seedlings with approximately 1-cm long roots were grown for 10 days in agar media that included 0 or 150 μM AlCl_3_ (0.5 mM CaCl_2_, pH 4.5). The samples were taken from three independent lines and equally mixed for quantitative analysis. The error bars indicate the standard errors of the means (SD) based on three technical replicates. The data are mean values ± SDs

**Fig. 9:**
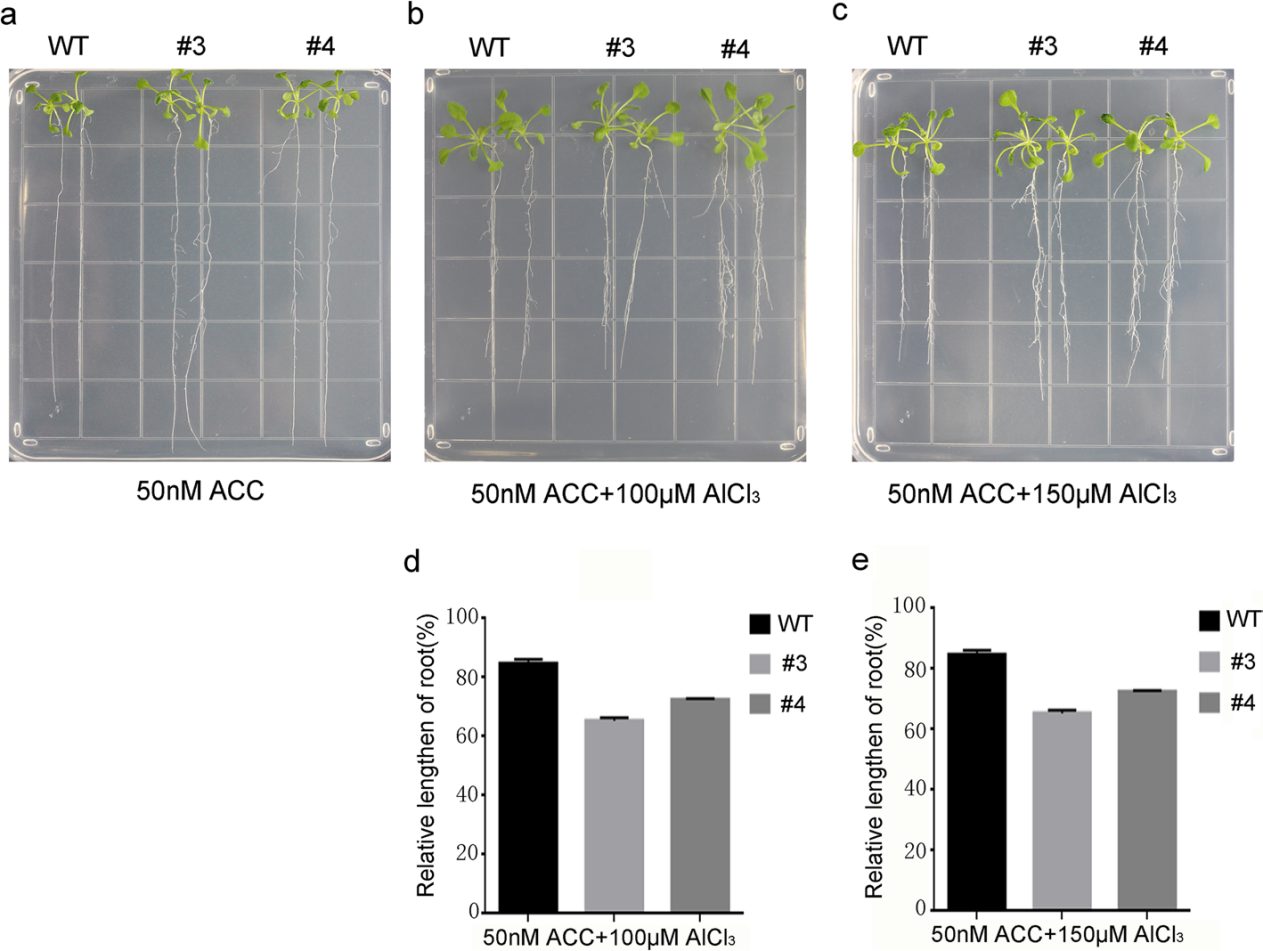
Phenotypes of GsERF1 transgenic plants in the presence of both ACC and aluminum. **a** Root growth of wild-type and *GsERF1*-overexpressing Arabidopsis plants under aluminum stress. **b **&** c**, Root growth of *GsERF1*-overexpressing and wild-type Arabidopsis under ACC and Al treatment. **d **&** e**, Relative root growth. Seedlings with approximately 1-cm-long roots were grown in agar media that included 50 nM ACC and 100 or 150 μM AlCl_3_ (0.5 mM CaCl_2_, pH 4.5) for 10 days. The error bars indicate the standard errors of the means (SD) based on three technical replicates. The data are mean values ± SDs

According to previous studies, plant hormones are involved in the response to stress. When plants are under stress, various hormones respond, and different hormones may interact to form a network of mutual exchange to resist external pressure [48]. In our study, we found that when GsERF1-overexpressing plants were subjected to aluminum stress, they produced more ethylene than the wild-type plants did. Specifically, transcription of the ethylene synthesis-related genes *ASC4*, *ASC5* and *ASC6* increased (Fig. 7). These results are similar to those found in other studies in which the *ASC1*, *ASC2*, and *ASC5* genes associated with ethylene synthesis increased under NaHCO_3_ stress in *GmERF7*-overexpressing lines [28]. Protein phosphatase 2A can reduce the toxicity of cadmium by regulating ethylene production in Arabidopsis, and ASC2 and ASC6 were found to be upregulated under cadmium stress [49]. In the present study, the root elongation advantage of transgenic Arabidopsis no longer occurred when ACC was added to the aluminum treatment solution (Fig. 9). We speculated that, within a certain range, ethylene levels may promote root elongation. However, the speculation by which endogenous ethylene levels within a certain range in plants under aluminum stress could have a positive effect on plant growth needs to be further verified. In addition, the content of abscisic acid was also measured to explore its potential role in regulating the response to aluminum stress. The results showed that there was little difference in ABA content between the *GsERF1*-overexpressing lines and wild-type Arabidopsis plants (Fig. S4). However, some genes involved in the abscisic acid signaling pathway were found to exhibit varied expression levels in the *GsERF1*-overexpressing lines (Fig. 8). Under aluminum stress, the transcripts of *ABI1* and *ABI2* in the *GsERF1*-overexpressing lines were significantly lower than those in the wild type (Fig. 8). Previous studies have shown that *ABI1* and *ABI2* play key roles in abscisic acid signal transduction and act as negative regulators in abscisic acid signal transduction [50]. The transcription of *ABI4* and *ABI5*, which are positive regulators of abscisic acid signal transduction, in the *GsERF1*-overexpressing lines was higher than that in the WT. [51, 52] Furthermore, *RD29B* was significantly upregulated in the *GsERF1*-overexpressing lines (Fig. 8). *RD29B* is mainly involved in drought, salt stress and abscisic acid responses through independent abscisic acid pathways, resulting in higher permeability and stress tolerance of plants [53, 54]. These results suggested that the *GsERF1* gene may regulate plant tolerance to aluminum stress through the ET pathway and/or the interaction between ethylene and abscisic acid.

## Conclusions

In summary, the *GsERF1* gene, which encodes a soybean ERF transcription factor, was induced in response to aluminum stress, ethylene and ABA, and its expression was greatest in the roots. *GsERF1* overexpression enhanced the tolerance of the transgenic plants to aluminum stress. Molecular analysis indicated that enhanced resistance to aluminum stress might result an increase in the transcription of ACC biosynthesis-related genes and ABA-responsive and/or stress-responsive genes together with proline accumulation. Therefore, the results suggested that GsERF1 may enhance tolerance to aluminum stress mainly through an ethylene-mediated pathway.

## MATERIALS and METHODS

### Plant material and stress treatments

Seeds from the wild soybean line BW69 were sown in a growth chamber maintained at 28 °C/25 °C under 70% relative humidity and a 14 h light/10 h darkness photoperiod. BW69 is an Al-resistant *Glycine soja* line, identified by Zeng et al. in our laboratory [3]. The seeds were germinated in vermiculite, and seedlings of uniform growth were selected and cultivated in nutrient solution (pH 5.8) for three days; the solution was renewed daily. After 3 days, the seedlings transplanted into 0.5 mM CaCl_2_ (pH 4.5) solution were evaluated for their response to aluminum treatment [55]. For ethylene stress, hydroponically grown seedlings were placed in an airtight plexiglass chamber, and ethylene gas was released after 2 ml of 40% ethephon and 1 g of NaHCO_3_ were dissolved in 200 ml of H_2_O [23].

Seeds of Arabidopsis ecotype Columbia (Col-0) plants were germinated and grown in a growth chamber with the following conditions: 22–24 °C, 60% relative humidity, 100 mol photons m^− 2^ s^− 1^ light intensity, and a 16 h light/8 h darkness photoperiod. The seeds were sown and grown in potting media from germination to harvest. For analysis of gene expression in Arabidopsis, seeds were sown on 1/2-strength MS agar plates in darkness for 4 days at 4 °C and then placed in a growth chamber. After 10 days, whole plants were collected as samples, and each sample consisted of at least 10 plants.

### RNA isolation, cDNA synthesis and quantitative real-time PCR

Total RNA was isolated using TRIzol (Tiangen Biotech, Beijing, China), and cDNA synthesis was performed by the use of a PrimeScript RT Reagent Kit (Takara). QRT–PCR analyses were performed using SYBR Premix ExTaq™ II Mix (TaKaRa, Shiga, Japan). *Actin3* (GenBank accession No. NP_001276160.2) was used as a reference in wild soybean. The Arabidopsis housekeeping gene *actin* (GenBank accession No. NP_188508.1) was used as a reference in Arabidopsis. The data were analyzed with the 2^-ΔΔCT^ method [56], and the primers used for qRT PCR are listed in Supplementary Table S1.

### GsERF1 gene isolation and sequence analysis

The *GsERF1* gene was isolated from wild soybean line BW69. The full sequence of *GsERF1* was amplified via PCR in conjunction with the following primer pair: 5′ – GGATCACGCCTCAAGTT − 3′ and 5′- CGAACCCTAAATCATCAG − 3′. The PCR products were inserted into the multiple cloning site of a pLB vector (Tiangen Biotech, Beijing, China), and the positive clones were sent for sequencing. Multiple sequence alignment analysis was performed using DNAMAN software. Homology analysis of *GsERF1* and the other 44 reference ERF superfamily genes was performed using MEGA 6.0 software through a neighbor-joining method. The amino acid sequences were obtained from GenBank (http://www.ncbi.nlm.nih.gov/genbank/) and Phytozome (http://phytozome.jgi.doe.gov/pz/portal.html).

### Subcellular localization analysis

To analyze the subcellular localization of the GsERF1 protein, full-length *GsERF1* was inserted into the *Nco*I/*Spe*I sites of a pCAMBIA1302 vector to generate a GsERF1-eGFP construct. The pCAMBIA1302-GsERF1-eGFP fusion construct was subsequently transformed into tobacco epidermal cells. After 2–3 days, the green fluorescence signals in tobacco epidermal cells were observed under a confocal laser-scanning microscope (Olympus FluoView FV1000, Japan). The excitation wavelengths used were 488 nm for eGFP and 580 nm for RFP, and the resolution was 600 dpi [57].

### In vitro transcriptional activation assays

For transactivation assays, the full-length *GsERF1* gene was inserted into the *Eco*RI/*Bam*HI sites of a pGBKT7 vector. The pGBKT7-GsERF1 construct was then transformed into yeast strain Y2H gold, and the transformants were grown on SD/−Trp media (Clontech) at 30 °C for 3 days. After selection of the yeast transformants carrying the *GsERF1* gene on SD (−Trp) media, they were transferred to SD (−Trp, X-α-Gal) media to evaluate their transcriptional activation. An empty plasmid was used as a negative control.

### Arabidopsis transformation and soybean hairy root transformation

Arabidopsis ecotype Col-0 was used for transformation. The full coding region of *GsERF1* driven by the CaMV 35S promotor was inserted into the plant expression binary vector pTF101.1, yielding pTF101.1-GsERF1. The construct was subsequently transformed into *Agrobacterium tumefaciens* strain GV3101, and then the target gene was transferred into Arabidopsis plants by the floral-dip method [58].

Five-day-old seedlings with unfolded cotyledons were used for soybean hairy root production. For the RNAi construct, 233 bp of the *GsERF1* coding region was cloned and inserted into a pMU103 vector. The overexpression vector and RNAi interference vector were then transferred into *A. rhizogenes* strain K599, after which the plants were transformed with the cells by hypocotyl injection [59]. An empty pTF101.1 plant expression binary vector was used as a control.

### Hematoxylin staining

The expression of the *GsERF1* gene in the hairy root lines was analyzed, and appropriate hairy root lines were selected for subsequent experiments. The hairy roots were treated with 0 or 25 μM AlCl_3_ (0.5 mM CaCl_2_, pH 4.5) for 6 h. After AlCl_3_ treatment, the hairy roots were washed three times with sterilized water and then stained with hematoxylin. The dyed roots were subsequently washed in sterile water for 30 minutes, after which they were observed and imaged through a Leica S8APO stereomicroscope (Leica, Germany) [46].

### Phenotypic analysis of Arabidopsis tolerance to aluminum stress

To analyze the phenotypes of *GsERF1*-overexpressing (OX) and wild-type (WT) Arabidopsis under aluminum stress, seeds of T_3_*GsERF1*-overexpressing and WT plants were used. Among them, three transgenic lines with high expression levels were selected. The seed surfaces were sterilized with 10% sodium hypochlorite for 10 minutes and subsequently washed with deionized water. The sterilized seeds were grown on 1/2-strength MS agar plates in darkness for 4 days at 4 °C. Then, the plates were oriented upright and placed in a growth chamber at 22–24 °C, a 60% relative humidity, a 100 μmol photons m-2 s-1 light intensity, and 16 h light/8 h darkness photoperiod. Seedlings with a root length of 1 cm were selected and transferred to 1/2-strength MS agar media (pH 4.5) with different AlCl_3_ concentrations. After 10 days, the length from the base of the rosette leaf to the tip of the taproot was measured with a ruler, and images were taken with a Canon EOS 750d camera [60].

### Physiological index assays

*GsERF1* overexpression and WT lines were treated with or without aluminum for 10 days, and whole plants were selected as samples. The free proline content was measured as described in detail previously [23]. The ethylene precursor (ACC) and abscisic acid contents were determined using an enzyme-linked immunosorbent assay (ELISA) [61].

### Statistical analysis

All experiments involving each group were performed at least in triplicate. The data are reported as the means ± SDs. All the data were analyzed via t tests by GraphPad Prism 6.01 software to assess significant differences between the means.

## Supplementary Information

**Additional file 1.**

**Additional file 2.**

**Additional file 3.**

## Data Availability

All the data used in this study are included in this published article and its additional files. The plant materials used in the current study are available from the corresponding author on reasonable request. Sequence data from this article can be found in the NCBI or phytozome database under the following accession numbers: TaERF3(ABQ52687.1), AtERF019(NC_003070.9), JERF1(NC_015443.3), TSRF1(NC_015446.3), SlERF36(NC_015447.3), TaPIEP1(ABU62817.1), AtERF5(AT5G47230), TdERF1(AY781352), AtERF74(AT1G53910), MsERF8(AEQ64868.1), JERF3(NC_015440.3), TERF1(NC_000008.11), AtERF9(AT5G44210), AtERF11(AT1G28370), AtERF53(AT2G20880), OsERF83(ABG00021.1), ERF1-V (ACN58181), OsERF71(XP_015643752.1), GmERF3(EU681278), GmERF7(NC_038243.1), GmERF75(Glyma10G016500), GsERF71(Glyma02g01960).

## Abbreviations

ERF: Ethylene response factor
ACC: 1-aminocyclopropane-1-carboxylic acid
ACS: ACC synthase
ABA: Abscisic acid
ABI: Abscisic acid insensitive
ET: Ethylene
WT: Wild type
OX: Overexpression

## References

[1] Zafar SA, Zaidi SS, Gaba Y (2020). Engineering abiotic stress tolerance via CRISPR/ Cas-mediated genome editing. J Exp Bot.

[2] Zheng SJ (2010). Crop production on acidic soils: overcoming aluminium toxicity and phosphorus deficiency. Ann Bot London.

[3] Zeng Q, Yang C, Ma Q, Li X, Dong W, Nian H (2012). Identification of wild soybean miRNAs and their target genes responsive to aluminum stress. BMC Plant Biol.

[4] Li CX, Yan JY, Ren JY (2020). A WRKY transcription factor confers aluminum tolerance via regulation of cell wall modifying genes. J Integr Plant Biol.

[5] Lou HQ, Fan W, Jin JF (2019). A NAC-type transcription factor confers aluminium resistance by regulating cell wall-associated receptor kinase 1 and cell wall pectin. Plant Cell Environ.

[6] Yamaji N, Huang CF, Nagao S (2009). A zinc finger transcription factor ART1 regulates multiple genes implicated in aluminum tolerance in Rice. Plant Cell.

[7] Pierik R, Sasidharan R, Voesenek LACJ (2007). Growth control by ethylene: adjusting phenotypes to the environment. J Plant Growth Regul.

[8] Herzog M, Striker GG, Colmer TD, Pedersen O (2016). Mechanisms of waterlogging tolerance in wheat--a review of root and shoot physiology. Plant Cell Environ.

[9] Cao WH, Liu J, Zhou QY (2006). Expression of tobacco ethylene receptor NTHK1 alters plant responses to salt stress. Plant Cell Environ.

[10] Thao NP, Khan MIR, Thu NBA (2015). Role of ethylene and its cross talk with other signaling molecules in plant responses to heavy metal stress. Plant Physiol.

[11] Masood A, Iqbal N, Khan NA (2012). Role of ethylene in alleviation of cadmium-induced photosynthetic capacity inhibition by Sulphur in mustard. Plant Cell Environ.

[12] Khan MIR, Khan NA (2014). Ethylene reverses photosynthetic inhibition by nickel and zinc in mustard through changes in PS II activity, photosynthetic nitrogen use efficiency, and antioxidant metabolism. Protoplasma.

[13] Khan MIR, Jahan B, AlAjmi MF, Rehman MT, Khan NA (2020). Ethephon mitigates nickel stress by modulating antioxidant system, glyoxalase system and proline metabolism in Indian mustard. Physiol Mol Biol Pla.

[14] Zhang G, Chen M, Chen X (2008). Phylogeny, gene structures, and expression patterns of the ERF gene family in soybean (Glycine max L.). J Exp Bot.

[15] Riechmann JL, Meyerowitz EM (1998). The AP2/EREBP family of plant transcription factors. Biol Chem.

[16] Hu Y, Zhao L, Chong K, Wang T (2008). Overexpression of OsERF1, a novel rice ERF gene, up-regulates ethylene-responsive genes expression besides affects growth and development in Arabidopsis. J Plant Physiol.

[17] Lee SY, Hwang EY, Seok HY (2015). Arabidopsis AtERF71/HRE2 functions as transcriptional activator via cis-acting GCC box or DRE/CRT element and is involved in root development through regulation of root cell expansion. Plant Cell Rep.

[18] Wessels B, Seyfferth C, Escamez S (2019). An AP2/ERF transcription factor ERF139 coordinates xylem cell expansion and secondary cell wall deposition. New Phytol.

[19] Jung H, Chung PJ, Park S (2017). Overexpression ofOsERF48 causes regulation ofOsCML16 , a calmodulin-like protein gene that enhances root growth and drought tolerance. Plant Biotechnol J.

[20] Jin Y, Pan W, Zheng X (2018). OsERF101, an ERF family transcription factor, regulates drought stress response in reproductive tissues. Plant Mol Biol.

[21] Li J, Guo X, Zhang M (2018). OsERF71 confers drought tolerance via modulating ABA signaling and proline biosynthesis. Plant Sci.

[22] Yu Y, Yang D, Zhou S (2017). The ethylene response factor OsERF109 negatively affects ethylene biosynthesis and drought tolerance in rice. Protoplasma.

[23] Zhang G, Chen M, Li L (2009). Overexpression of the soybean GmERF3 gene, an AP2/ERF type transcription factor for increased tolerances to salt, drought, and diseases in transgenic tobacco. J Exp Bot.

[24] Scarpeci TE, Frea VS, Zanor MI, Valle EM (2016). Overexpression ofAtERF019 delays plant growth and senescence and improves drought tolerance in Arabidopsis. J Exp Bot.

[25] Zhao M, Yin L, Ma J (2019). The roles of GmERF135 in improving salt tolerance and decreasing ABA sensitivity in soybean. Front Plant Sci.

[26] Zhai Y, Wang Y, Li Y (2013). Isolation and molecular characterization of GmERF7, a soybean ethylene-response factor that increases salt stress tolerance in tobacco. Gene.

[27] Xing L, Di Z, Yang W (2017). Overexpression of ERF1-V from Haynaldia villosa can enhance the resistance of wheat to powdery mildew and increase the tolerance to salt and drought stresses. Front Plant Sci.

[28] Yu Y, Liu A, Duan X (2016). GsERF6, an ethylene-responsive factor from Glycine soja, mediates the regulation of plant bicarbonate tolerance in Arabidopsis. Planta.

[29] Yu Y, Duan X, Ding X (2017). A novel AP2/ERF family transcription factor from Glycine soja, GsERF71, is a DNA binding protein that positively regulates alkaline stress tolerance in Arabidopsis. Plant Mol Biol.

[30] Li W, Wang C, Shi H (2020). Genome-wide analysis of ethylene-response factor family in adzuki bean and functional determination of VaERF3 under saline-alkaline stress. Plant Physiol Bioch.

[31] Yu F, Liang K, Fang T (2019). A group VII ethylene response factor gene, ZmEREB180, coordinates waterlogging tolerance in maize seedlings. Plant Biotechnol J.

[32] Sun X, Zhang L, Wong DCJ (2019). The ethylene response factor VaERF092 from Amur grape regulates the transcription factor VaWRKY33, improving cold tolerance. Plant J.

[33] Bolt S, Zuther E, Zintl S, Hincha DK, Schmülling T (2017). ERF105 is a transcription factor gene ofArabidopsis thaliana required for freezing tolerance and cold acclimation. Plant Cell Environ.

[34] Zhao M, Yin L, Liu Y (2019). The ABA-induced soybean ERF transcription factor gene GmERF75 plays a role in enhancing osmotic stress tolerance in Arabidopsis and soybean. BMC Plant Biol.

[35] Oñate-Sánchez L, Anderson JP, Young J, Singh KB (2007). AtERF14, a member of the ERF family of transcription factors, plays a nonredundant role in plant defense. Plant Physiol.

[36] Zheng X, Xing J, Zhang K (2019). Ethylene response factor ERF11 ActivatesBT4 transcription to regulate immunity toPseudomonas syringae. Plant Physiol.

[37] Zhang H (2015). Arabidopsis AtERF15 positively regulates immunity against pseudomonas syringae pv. Tomato DC3000 and Botrytis cinerea. Front Plant Sci.

[38] Dong L, Cheng Y, Wu J (2015). Overexpression of GmERF5, a new member of the soybean EAR motif-containing ERF transcription factor, enhances resistance to Phytophthora sojae in soybean. J Exp Bot.

[39] Zhao Y, Chang X, Qi D (2017). A novel soybean ERF transcription factor, GmERF113, increases resistance to Phytophthora sojae infection in soybean. Front Plant Sci.

[40] Klay I, Gouia S, Liu M (2018). Ethylene response factors (ERF) are differentially regulated by different abiotic stress types in tomato plants. Plant Sci.

[41] Sohaib Ahmed MARR, Zafar MWMU, Tehseen Azhar IARF, Chung ZARM (2020). Genome-wide investigation and expression analysis of APETALA-2 transcription factor subfamily reveals its evolution, expansion and regulatory role in abiotic stress responses in Indica Rice (Oryza sativa L. ssp. indica). Genomics.

[42] An JP, Zhang XW, Bi SQ, You CX, Wang XF, Hao YJ (2019). The ERF transcription factor MdERF38 promotes drought stress-induced anthocyanin biosynthesis in apple. Plant J.

[43] Wang M, Dai W, Du J, Ming R, Dahro B, Liu JH (2019). ERF 109 of trifoliate orange (Poncirus trifoliata (L.) Raf.) contributes to cold tolerance by directly regulating expression of Prx1 involved in antioxidative process. Plant Biotechnol J.

[44] Wang X, Liu S, Tian H, Wang S, Chen J (2015). The small ethylene response factor ERF96 is involved in the regulation of the abscisic acid response in Arabidopsis. Front Plant Sci.

[45] Zhang J, Xu H, Wang N (2018). The ethylene response factor MdERF1B regulates anthocyanin and proanthocyanidin biosynthesis in apple. Plant Mol Biol.

[46] Rincón M, Gonzales RA (1992). Aluminum partitioning in intact roots of aluminum-tolerant and aluminum-sensitive wheat (Triticum aestivum L.). Cultivars Plant Physiol.

[47] Wang J, Raman H, Zhang G, Mendham N, Zhou M (2006). Aluminium tolerance in barley (Hordeum vulgare L.): physiological mechanisms, genetics and screening methods. J Zhejiang Univ Sci B.

[48] Ku Y, Sintaha M, Cheung M, Lam H (2018). Plant hormone signaling Crosstalks between biotic and abiotic stress responses. Int J Mol Sci.

[49] Chen J, Wang X, Zhang W, Zhang S, Zhao FJ (2020). Protein phosphatase 2A alleviates cadmium toxicity by modulating ethylene production inArabidopsis thaliana. Plant Cell Environ.

[50] Seo YJ, Park J, Cho Y (2010). Overexpression of the ethylene-responsive factor gene BrERF4 from Brassica rapa increases tolerance to salt and drought in Arabidopsis plants. Mol Cells.

[51] Finkelstein RR, Wang ML, Lynch TJ, Rao S, Goodman HM (1998). The Arabidopsis abscisic acid response locus ABI4 encodes an APETALA2 domain protein. Plant Cell.

[52] Finkelstein RR, Lynch TJ (2000). The Arabidopsis abscisic acid response gene ABI5 encodes a basic leucine zipper transcription factor. Plant Cell.

[53] Shinozaki KYAK. A Nove1 cis-acting element in an Arabidopsis gene 1s lnvolved in responsiveness to drought, Lowqemperature, or high-salt stress. Labor Plant Mol Biol. 1994;6(2):251–64. 10.1105/tpc.6.2.251 PMC160431 8148648

[54] Msanne J, Lin J, Stone JM, Awada T (2011). Characterization of abiotic stress-responsive Arabidopsis thaliana RD29A and RD29B genes and evaluation of transgenes. Planta.

[55] Zhu XF, Sun Y, Zhang BC (2014). TRICHOME BIREFRINGENCE-LIKE27 affects aluminum sensitivity by modulating the O-acetylation of xyloglucan and aluminum-binding capacity in Arabidopsis. Plant Physiol.

[56] Livak KJ, Schmittgen TD (2001). Analysis of relative gene expression data using real-time quantitative PCR and the 2−ΔΔCT method. Methods.

[57] Sparkes IA, Runions J, Kearns A, Hawes C (2006). Rapid, transient expression of fluorescent fusion proteins in tobacco plants and generation of stably transformed plants. Nat Protoc.

[58] CLOUGH SJ. (2010). Floral dip : a simplified method for Agrobacterium-mediated transformation of Arabidopsis thaliana. Plant J.

[59] Guo W, Zhao J, Li X, Qin L, Yan X, Liao H (2011). A soybean β-expansin gene GmEXPB2 intrinsically involved in root system architecture responses to abiotic stresses. Plant J.

[60] Ma Q, Yi R, Li L (2018). GsMATE encoding a multidrug and toxic compound extrusion transporter enhances aluminum tolerance in Arabidopsis thaliana. BMC Plant Biol.

[61] Yang J, Zhang J, Wang Z, Zhu Q, Wang W (2001). Hormonal changes in the grains of Rice subjected to water stress during grain Filling1. Plant Physiol.

