# Supplementary material for: GsERF1 enhances Arabidopsis thaliana aluminum tolerance through an ethylene-mediated pathway
Source: BMC Plant Biol. 2022 May 24;22:258. doi: 10.1186/s12870-022-03625-6 (PMC9128276; doi:10.1186/s12870-022-03625-6)
Supplement: Supplementary file 1 — Additional file 1. [file 12870_2022_3625_MOESM1_ESM.docx]

Additional files 1.


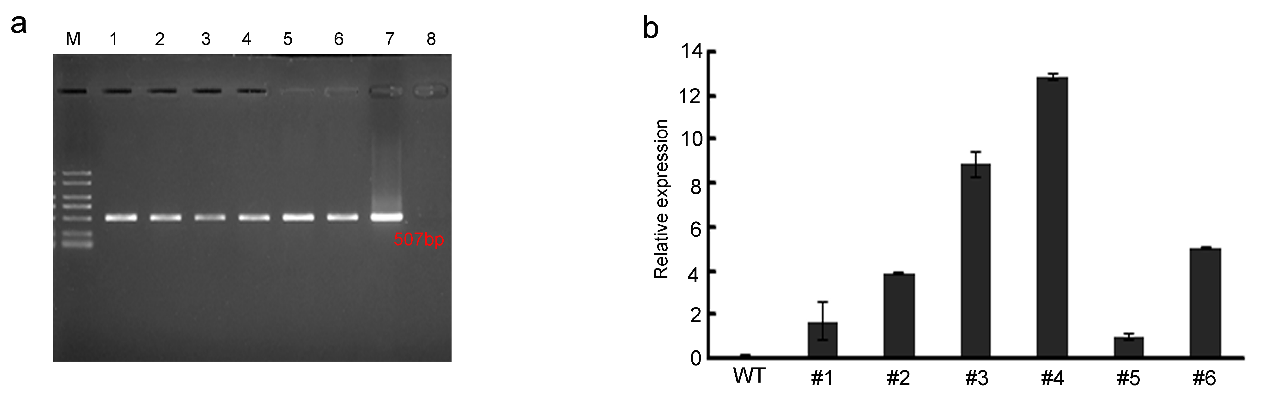


Figure S1. Molecular identification for *GsERF1* transgenic Arabidopsis lines.

a, PCR identification of *GsERF1* transgenic plants. M: DNA maker DL2000; Lane 1-8: PCR products with different DNA templates. Set as plasmid for lane 7, genomic DNA from wide type for lane 8, genomic DNA from *GsERF1* transgenic plants for lane 1-6. b, qRT-PCR identification of *GsERF1* transgenic lines. WT: wild type of Arabidopsis Columbia-0; #1 to #6: six transgenic lines of *GsERF1* in T_3_ generation. Data are means ± SD. Error bars represent the standard error of three replicates.

Additional files 2


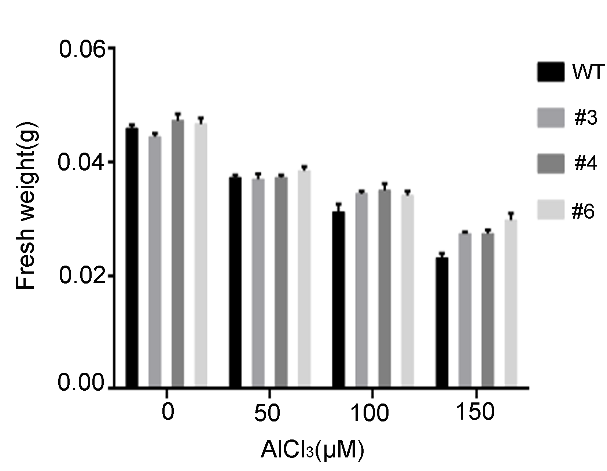


Figure S2. Fresh weight of GsERF1-overexpressing and wild type Arabidopsis after aluminum treatment.

Additional files 3


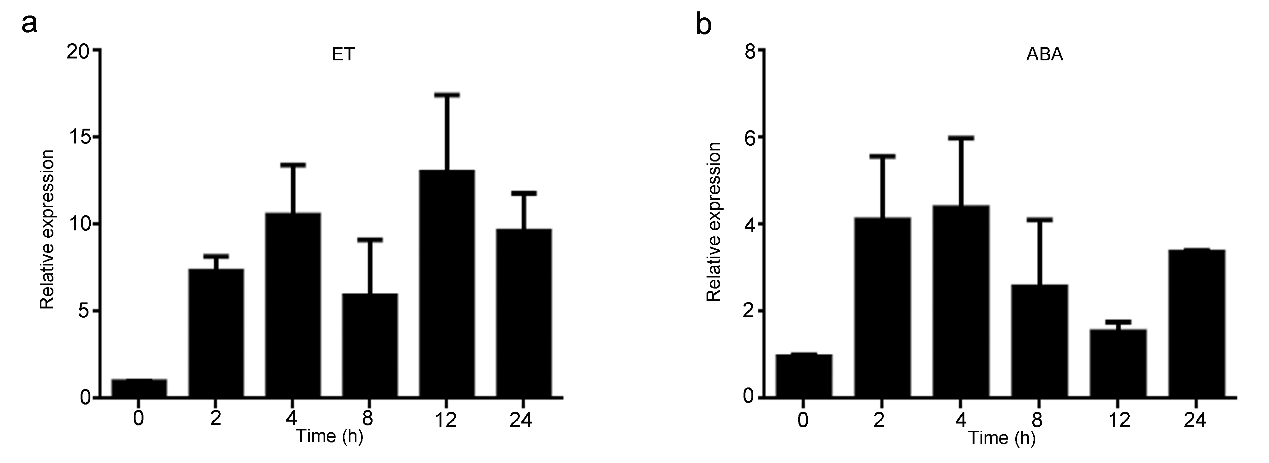


Figure S3. The relative expression of *GsERF1* in the BW69 line of *Glycine soja* under ethylene and abscisic acid treatments. Error bars indicate standard error of the means (SD) based on three technical replicates. Data are mean values ± SD.

Additional files 4


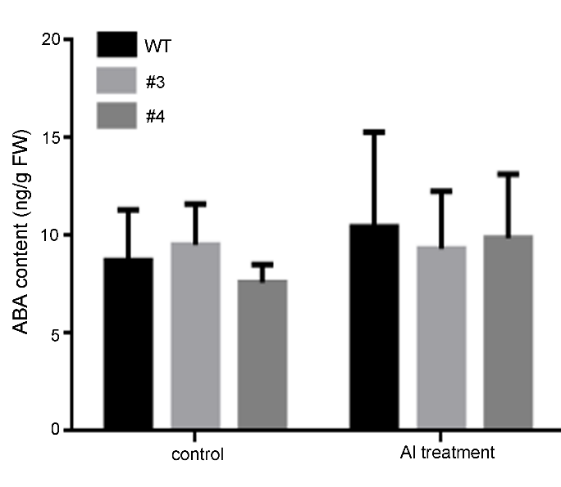


Figure S4. ABA content in GsERF1-overexpressing and wild type Arabidopsis. Data are mean values ± SD. Error bars represent the standard error of three replicates. WT: wild type. #3 and #4: transgenic lines of GsERF1 in T3 generation.

Additional files 5


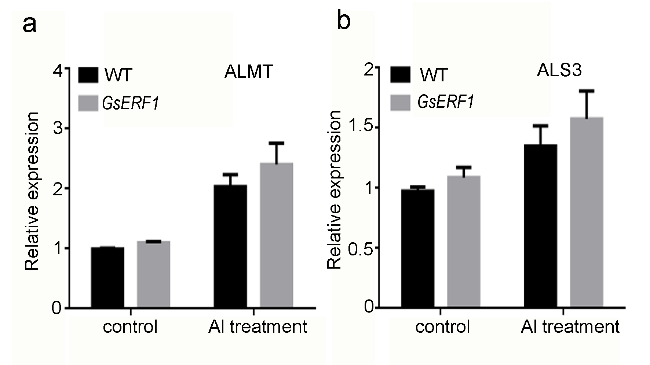


Figure S5. The expression of resistance gene to aluminum stress. a, AtALMT1expression. b, AtALS3expression. Seedlings with approximately 1-cm roots were grown in agar medium containing 0 or 150 mM AlCl3 for 10 days. The samples were taken from three independent lines and equally mixed for the quantitative analysis. Error bars indicate standard error of the means (SD) based on three technical replicates. Data are mean values ± SD.
