# Supplementary material for: GsERF1 enhances Arabidopsis thaliana aluminum tolerance through an ethylene-mediated pathway
Source: BMC Plant Biol. 2022 May 24;22:258. doi: 10.1186/s12870-022-03625-6 (PMC9128276; doi:10.1186/s12870-022-03625-6)
Supplement: Supplementary file 2 — Additional file 2. [file 12870_2022_3625_MOESM2_ESM.docx]

Additional file 6

Sequence of coding region of GsERF1 gene： ATGGAGAAAGAGAGAGGAGAGGAAGAAGTGAAGTACCGTGGAGTGAGAAAGAGGCCATGGGGTAAGTTCGGAGCAGAGATCAGAGACCCAACAAAACCTACGGGAAGGCAATGGTTAGGGACATTTGACACTGCTGAAGAAGCTGCTAGAGCTTATGATCGTGCAGCCATTGCTTTGAGGGGTGCTCTTGCAATCCTTAATTTTCCTCATGAGTTCCATTCTCATCTCCCTTTTATATTATCAAATTCTTCTACTAAGGGAAATGGAAGTTCTTTTGATAAGGAAGTTATTGAGTTAGAGTATTTGGATGACAAGGTGTTGGAAGAGCTTCTTGAGTTAGAAGAGAAGAGAAGGAATAACGAAGACTAA
