# Supplementary material for: GsERF1 enhances Arabidopsis thaliana aluminum tolerance through an ethylene-mediated pathway
Source: BMC Plant Biol. 2022 May 24;22:258. doi: 10.1186/s12870-022-03625-6 (PMC9128276; doi:10.1186/s12870-022-03625-6)
Supplement: Supplementary file 3 — Additional file 3. [file 12870_2022_3625_MOESM3_ESM.docx]

| Table S1 List of the primers in present study | | |
| --- | --- | --- |
| Primer name | Purpose | Primer sequence 5’ to 3’ |
| *GsERF1*-F | GsERF1 cloning | GGATCACGCCTCAAGTT |
| *GsERF1*-R |  | CGAACCCTAAATCATCAG |
| qERF1-F | qRT-PCR | CAAATTCTTCTACTAAGGGAAATGG |
| qERF1-R |  | GTCTTCGTTATTCCTTCTCTTCTC |
| ACT3-F | qRT-PCR | GCACCACCGGAGAGAAAATA |
| ACT3-R |  | GTGCACAATTGATGGACCAG |
| pTF101-czF | pTF101.1-GsERF1 | GAGAACACGGGGGACTCTAGAATGGAGAAAGAGAGAGGAGAGGAAG |
| pTF101-czR |  | CGATCGGGGAAATTCGAGCTCTTAGTCTTCGTTATTCCTTCTCTTCTCT |
| pGBTK7CZ-F | pGBTK7-GsERF1 | atggccatggaggccgaattcATGGAGAAAGAGAGAGGAGAGGAAG |
| pGBTK7CZ-R |  | ccgctgcaggtcgacggatccCTTAGTCTTCGTTATTCCTTCTCTTCTCT |
| 1302CZ-F | P1302-eGFP-GsERF1 | acgggggactcttgaccatggCTATGGAGAAAGAGAGAGGAGAGGAAG |
| 1302CZ-R |  | aagttcttctcctttactagtGTCTTCGTTATTCCTTCTCTTCTCTTC |
| Bar-F | Molecular identification for GsERF1 transgenic lines by bar gene | AAACCCACGTCATGCCAGT |
| Bar-R |  | TCTGCACCATCGTCAACCAC |
| Actin-F | qRT-PCR | TTACCCGATGGGCAAGTC |
| Actin-R |  | GCTCATACGGTCAGCGATAC |

| Table S2 List of gene id | |
| --- | --- |
| Gene name | Gene ID |
| JERF1 | NC_015443.3 |
| AtERF5 | AT5G47230 |
| AtERF7 | AT3G20310 |
| AtERF8 | AT1G53170 |
| AtERF9 | AT5G44210 |
| AtERF11 | AT1G28370 |
| AtERF14 | AT1G04370 |
| AtERF15 | AT2G31230 |
| AtERF019 | NC_003070.9 |
| AtERF71 | AT2G47520 |
| AtERF72 | AT3G16770 |
| AtERF74 | AT1G53910 |
| AtERF96 | AT5G43410 |
| AtERF105 | AT5G51190 |
| AtERF53 | AT2G20880 |
| ERF1-V | ACN58181 |
| GmERF3 | EU681278 |
| GmERF5 | NC_038250.1 |
| GmERF7 | NC_038243.1 |
| GmERF75 | NC_038246.1 |
| GmERF113 | NC_038252.1 |
| GmERF135 | NC_038253.1 |
| JcERF2 | NW_012125068.1 |
| JERF3 | NC_015440.3 |
| MsERF8 | AEQ64868.1 |
| OsERF71 | XP_015643752.1 |
| OsERF83 | ABG00021.1 |
| SlERF36 | NC_015447.3 |
| TaERF3 | ABQ52687.1 |
| TaPIEP1 | ABU62817.1 |
| TdERF1 | AY781352 |
| TERF1 | NC_000008.11 |
| TSRF1 | NC_015446.3 |
